# Validity of Electronic Diet Recording Nutrient Estimates Compared to Dietitian Analysis of Diet Records: Randomized Controlled Trial

**DOI:** 10.2196/jmir.3744

**Published:** 2015-01-20

**Authors:** Susan K Raatz, Angela J Scheett, LuAnn K Johnson, Lisa Jahns

**Affiliations:** ^1^United States Department of Agriculture, Agricultural Research ServiceGrand Forks Human Nutrition Research CenterGrand Forks, NDUnited States; ^2^University of MinnesotaDepartment of Food Science and NutritionMinneapolis, MNUnited States; ^3^University of North DakotaGrand Forks, NDUnited States

**Keywords:** diet records, nutrition assessment, electronic data

## Abstract

**Background:**

Dietary intake assessment with diet records (DR) is a standard research and practice tool in nutrition. Manual entry and analysis of DR is time-consuming and expensive. New electronic tools for diet entry by clients and research participants may reduce the cost and effort of nutrient intake estimation.

**Objective:**

To determine the validity of electronic diet recording, we compared responses to 3-day DR kept by Tap & Track software for the Apple iPod Touch and records kept on the Nutrihand website to DR coded and analyzed by a research dietitian into a customized US Department of Agriculture (USDA) nutrient analysis program, entitled GRAND (Grand Forks Research Analysis of Nutrient Data).

**Methods:**

Adult participants (n=19) enrolled in a crossover-designed clinical trial. During each of two washout periods, participants kept a written 3-day DR. In addition, they were randomly assigned to enter their DR in a Web-based dietary analysis program (Nutrihand) or a handheld electronic device (Tap & Track). They completed an additional 3-day DR and the alternate electronic diet recording methods during the second washout. Entries resulted in 228 daily diet records or 12 for each of 19 participants. Means of nutrient intake were calculated for each method. Concordance of the intake estimates were determined by Bland-Altman plots. Coefficients of determination (*R* ^2^) were calculated for each comparison to assess the strength of the linear relationship between methods.

**Results:**

No significant differences were observed between the mean nutrient values for energy, carbohydrate, protein, fat, saturated fatty acids, total fiber, or sodium between the recorded DR analyzed in GRAND and either Nutrihand or Tap & Track, or for total sugars comparing GRAND and Tap & Track. Reported values for total sugars were significantly reduced (*P*<.05) comparing Nutrihand to GRAND. Coefficients of determination (*R* ^2^) for Nutrihand and Tap & Track compared to DR entries into GRAND, respectively, were energy .56, .01; carbohydrate .58, .08; total fiber .65, .37; sugar .78, .41; protein .44, .03; fat .36, .03; saturated fatty acids .23, .03; sodium .20, .00; and for Nutrihand only for cholesterol .88; vitamin A .02; vitamin C .37; calcium .05; and iron .77. Bland-Altman analysis demonstrates high variability in individual responses for both electronic capture programs with higher 95% limits of agreement for dietary intake recorded on Tap & Track.

**Conclusions:**

In comparison to dietitian-entered 3-day DR, electronic methods resulted in no significant difference in mean nutrient estimates but exhibited larger variability, particularly the Tap & Track program. However, electronic DR provided mean estimates of energy, macronutrients, and some micronutrients, which approximated those of the dietitian-analyzed DR and may be appropriate for dietary monitoring of groups. Electronic diet assessment methods have the potential to reduce the cost and burden of DR analysis for nutrition research and clinical practice.

**Trial Registration:**

Clinicaltrials.gov NCT01183520; http://clinicaltrials.gov/ct2/show/NCT01183520 (Archived by WebCite at http://www.webcitation.org/6VSdYznKX).

## Introduction

Assessment of food intake to determine nutrient consumption in free living conditions is an integral part of dietetics practice and nutrition research. These data are essential for evaluating intake and making client-specific nutritional recommendations in clinical practice [1]. Additionally, intake assessment is an integral part of nutrition research as nutrient intake is often the primary or secondary endpoint in human trials [2]. The collection and analysis of dietary records (DR) is a standard tool for the evaluation of nutrient intake in both the clinical and research settings and is used extensively by dietitians and nutritionists. Furthermore, dietary record keeping is an important tool for patients/clients to self-monitor their progress with weight loss or their management of diet-related diseases such as diabetes. The availability of diet assessment methods with low respondent burden coupled with reliable food composition data in nutrient analysis programs is essential for monitoring nutrient intake of groups and individuals, to evaluate nutritional advice compliance and to conduct nutrition research [3].

Typically patients, clients, or study participants are taught to record their intake for a specified number of consecutive days, with record length of ≥3 days deemed as appropriate for usual diet representation [4]. Absent biomarkers, the DR is often considered the “gold standard” for determination of actual food intake and is generally utilized as the evaluation tool for determining the validity of other dietary assessment methods [5,6].

Manual entry of hand-recorded DR into a nutrient analysis program must be performed to generate the desired nutrient value output. Qualified personnel (typically dietitians, nutritionists, or technical staff under their supervision) perform DR entry. The collection of records, coding, and entry into nutrient analysis programs, and generation of desired data is costly in terms of personnel time and effort [7]. Valid, reliable, and inexpensive diet assessment tools with low time investment for both clients and dietitians are required to reduce the labor and financial costs to clinicians and researchers [8-10].

Depending on the purpose of the nutrient data collection, more automated methods of diet recording may prove useful. Many applications are available for use on the Web, for personal computing, and for handheld devices such as mobile phones [11]. In clinical nutrition practice, DRs are used to evaluate nutritional adequacy and to monitor dietary change after counseling. Nutrient intake data obtained in research projects may serve as the primary or secondary endpoints assessing intake or determining compliance and/or diet change. Therefore, careful evaluation of electronic diet recording methods is required to evaluate the validity of their use in clinical practice and research.

DR may be more efficiently entered by clients or research participants with electronic methods rather than hand-recorded. Electronic tools that are currently available for DR have the potential to reduce effort spent in diet entry by the dietitian. The utility of commercial methods of electronic diet capture has not been widely studied; the validity of such methods must be ascertained to demonstrate their suitability for clinical and/or research data collection. Herein, we report a study performed to assess the validity of two electronic DR methods compared with dietitian-coded, handwritten DR in a sample of 19 healthy volunteers. We compared nutrient analysis data of 3-day DR kept by Tap & Track (software [12] for the Apple iPod Touch), records entered into the Nutrihand website [13], and those coded and entered by a dietitian into a customized US Department of Agriculture (USDA) nutrient analysis program.

## Methods

### Study Design

A total of 19 participants (11 women, 8 men), enrolled in a crossover-designed clinical trial evaluating intake of farmed Atlantic salmon, are included in this experiment [14]. All participants were recruited from the Greater Grand Forks Area, Grand Forks, ND. The mean age of the group was 51.6 (SE 1.5) years and the mean body mass index was 29.2 (SE 0.6) kg/m^2^.

All participants completed three dietary treatments of 4 weeks, each separated by a 4-week washout period. During each of the washout periods, participants maintained 3-day DR by handwritten record and by electronic capture with random assignment to one of the two electronic tools under evaluation. During the second washout period, both the alternate electronic DR and an additional 3-day DR were obtained. These entries resulted in 228 daily DR for statistical analysis.

The study was approved by the Institutional Review Board of the University of North Dakota. Informed consent was obtained from all study participants prior to initiation of the study. The trial was registered at clinicaltrials.gov as NCT01183520.

### Dietary Measurement Tools

All participants were provided in-depth, individual instruction from a research dietitian on how to maintain a DR, how to access Nutrihand, the Web-based program, and how to use the iPod-based Tap & Track program. DR instruction was provided by a research dietitian at the USDA, Agricultural Research Service (ARS), Grand Forks Human Nutrition Research Center (GFHNRC). Standard forms were provided for recording purposes, which included entry of food items, descriptions, and amounts consumed. Participants were advised to record food consumption at each eating occasion. The need for detailed descriptions of foods and beverages consumed was emphasized and the dietitian instructed participants to estimate portions consumed using household measures (eg, measuring cups or spoons), serving size weight from a Nutrition Facts Label, number, consumed actual weight of item from a food scale, or Food Intake and Analysis System (FIAS) 2-dimensional food models [5].

Diet records were obtained from November, 2010 to May, 2011. Participants submitted their written DR to the dietitian and were interviewed to assure completeness of the records. Participants were queried about the portion sizes, exact food items, and additions of condiments to foods consumed. The dietitian coded and entered the records obtained at each washout period into the GFHNRC customized in-house nutrient analysis program. In addition, during each washout period, participants were randomly assigned to record their diet concurrently in one of the electronic apps: Tap & Track software (Nanobit Software, version 4.9.8), which is an app for the Apple iPod Touch, or on the Web using Nutrihand.

Client-recorded DR were reviewed and coded by the study dietitian (AS) and entered into the nutrient analysis program entitled GRAND (Grand Forks Research Analysis of Nutrient Data). GRAND is the customized nutrient analysis program of the GFHNRC using the USDA National Nutrient Database for Standard Reference, Release 22 (SR22) for nutrient values [15].

Tap & Track was selected for use due to its ability to collect data without connection to the Internet. This app is specifically marketed for use by individuals to monitor their intake and was one of the highest consumer-rated diet assessment apps in the iTunes App Store at the time this study was planned. Study participants were trained to use this app on an Apple iPod Touch provided to them for study purposes. Current versions of this app are available for iPhone use. Participants were asked to search the database for reported foods and beverages. Completed logs listing foods consumed and their nutrient values were downloaded from the instrument into a spreadsheet.

Nutrihand was selected for use due to the ability of a dietitian to assign clients and view client-entered DR. The program is marketed to the dietitian and other health professionals as a monitoring tool for use with clients. A HIPAA-compliant sign-on allows the dietitian to both monitor entries and communicate with clients. In addition to the food intake monitoring, additional functions such as physical activity monitoring and medical data recording are part of the program. For the purposes of this trial, only the dietary record-keeping function was utilized. Participants were trained to use this website on a personal computer with Internet access. They were asked to search the database for reported foods and beverages. Nutrihand records were analyzed with their database, which used the USDA National Nutrient Database for Standard Reference (Release 21 as of 9/2013), along with recipes, branded foods, restaurant information, and items entered by users from nutrition fact labels or as recipes. Reports from completed DR were generated and downloaded from the website as a text file for study comparison.

### Statistical Analysis

All statistical analyses were performed in SAS (version 9.3, SAS Institute Inc., Cary, NC). Means and standard deviations for macro- and micronutrients were calculated from the DR entered into the GRAND, Tap & Track, and Nutrihand programs. Mixed model analysis of variance was used to test for differences between methods and whether observed differences were dependent upon the order in which the recording methods (Nutrihand or Tap & Track) were used. Method and time were fixed effects and subjects were random effects. Concordance of results obtained from dietitian entry of DR into GRAND and each electronic diet recording method were evaluated using Bland-Altman plots [16,17] to determine agreement between the two measures. In these plots, the difference between values obtained from two methods (electronic method vs GRAND) is plotted against the average of the values. The limits of agreement between the two methods are calculated as the mean of the differences±1.96∗(standard deviation of the differences) for each nutrient by each method of comparison (electronic methods vs GRAND). To assess the strength of the linear relationship between methods, correlations were computed between the macro- and micronutrient estimates obtained from Tap & Track and Nutrihand to those obtained from GRAND. Coefficients of determination, *R* ^2^, are reported for each comparison. Linear regression lines are shown in the figures to assist in visualizing the relationships between the methods.

## Results

All participants completed the hand-recorded DR and the electronic dietary records to which they were assigned during each washout period of the feeding trial. Participants reported 114 matched days of DR, Nutrihand, and Tap & Track (n=228 recalls) from records obtained on 3 consecutive days including 2 weekdays and 1 weekend day (either Thursday, Friday, and Saturday or Sunday, Monday, and Tuesday).

Table 1 includes the nutrient values obtained by each DR assessment method. The nutrient data available were more limited in the electronic capture methods than the GRAND program. No significant differences were observed between the mean nutrient values obtained for energy, carbohydrate, protein, fat, saturated fatty acids, total fiber, or sodium between GRAND and either Nutrihand or Tap & Track, and for total sugars comparing GRAND and Tap & Track. Reported values for total sugars were all significantly reduced (*P*<.05) comparing Nutrihand to GRAND.

**Figure 1:**
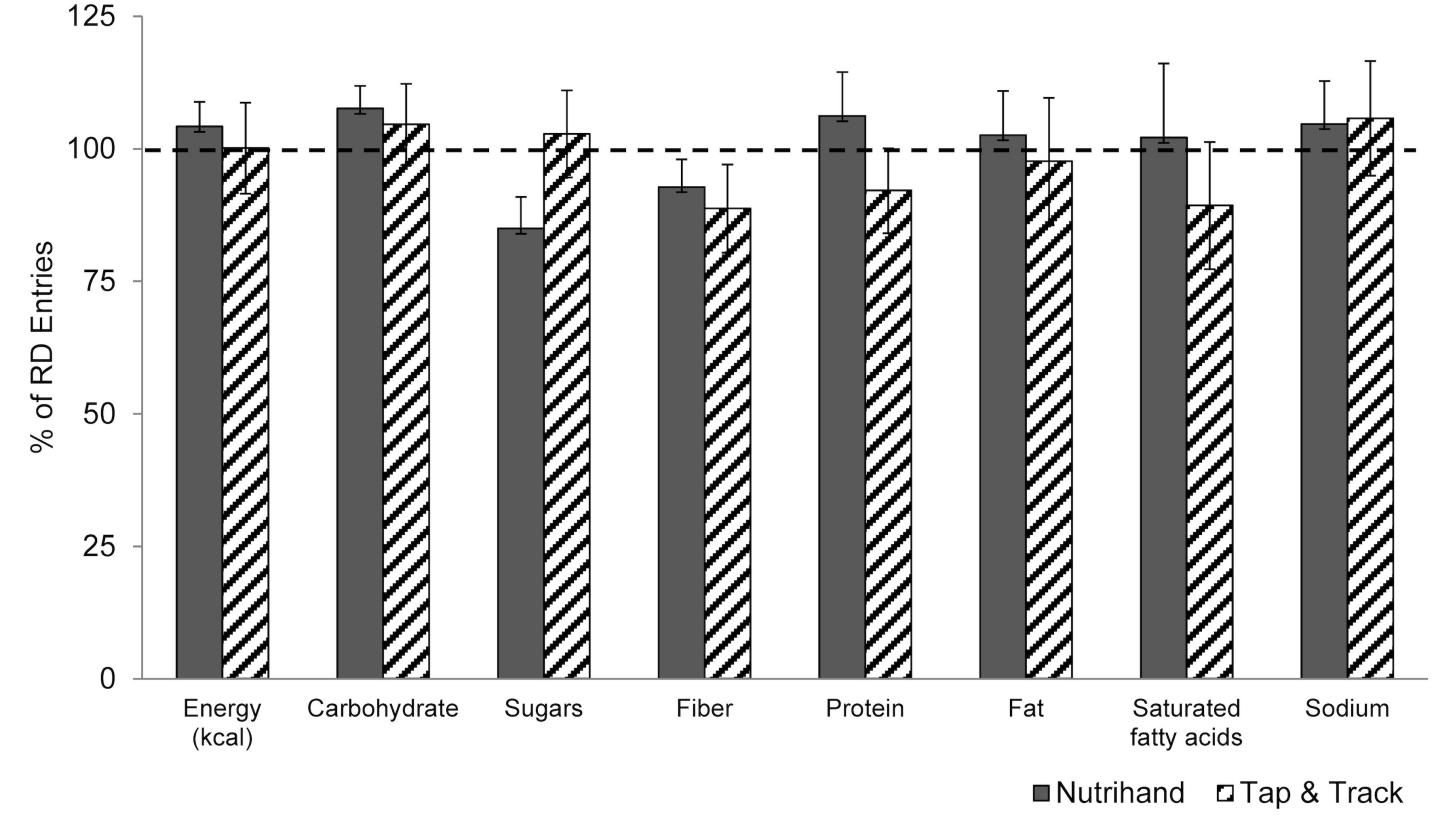
Percentage agreement between electronic methods of diet recording and dietitian-entered 3-day diet record (DR) comparing Nutrihand and Tap & Track to values obtained from Grand Forks Research Analysis of Nutrient Data (GRAND).

**Figure 2:**
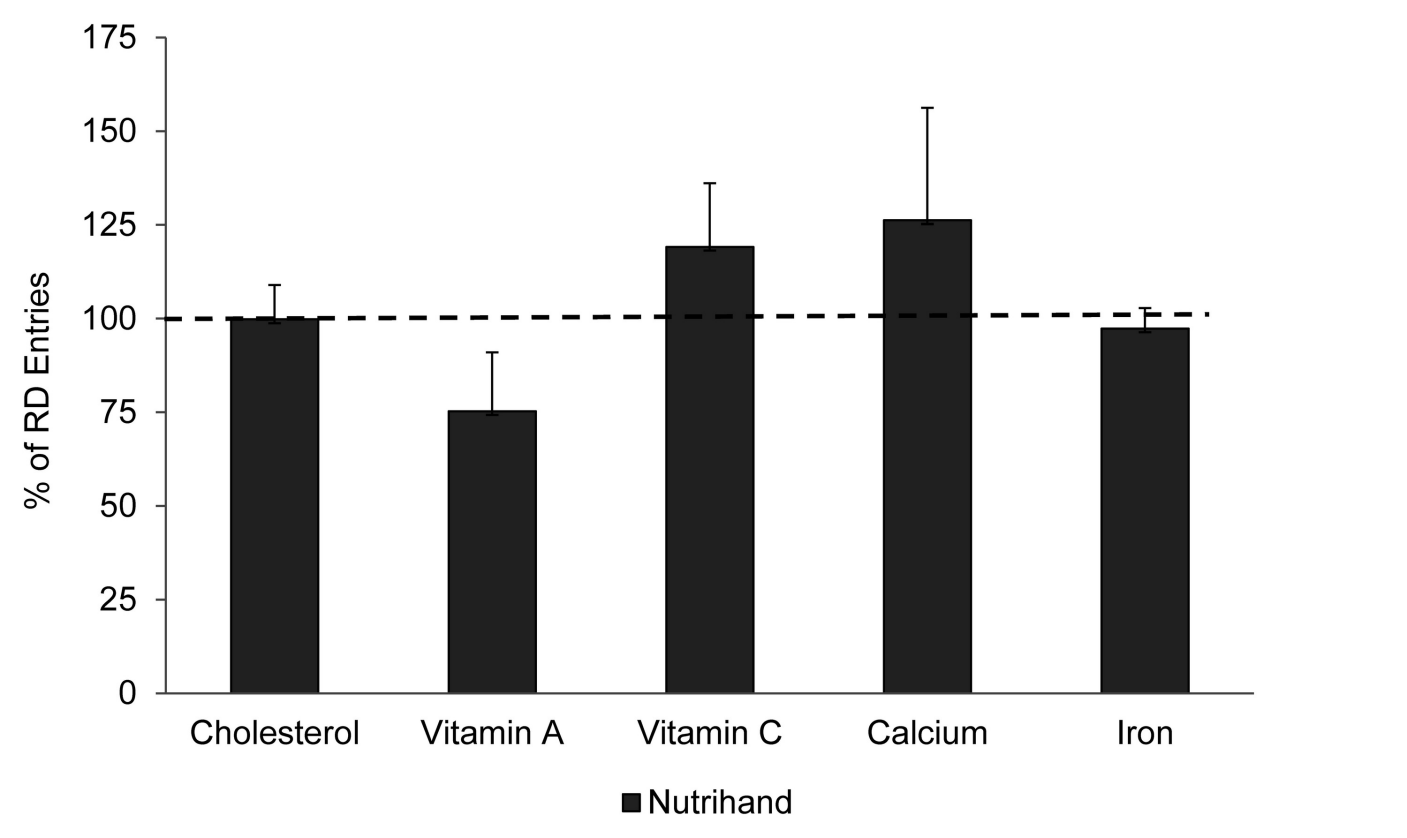
Percentage agreement between electronic methods of diet recording and dietitian-entered 3-day diet record (DR) comparing Nutrihand to values obtained from Grand Forks Research Analysis of Nutrient Data (GRAND).

Figures 1 and 2 illustrate the percentage of values obtained electronically compared to dietitian entry of DR into GRAND. Values were as follows (mean %, SE; Nutrihand and Tap & Track, respectively): energy 104.2 (SE 4.7), 100.1 (SE 8.6); carbohydrate 107.6 (SE 4.3), 104.6 (SE 7.7); sugar 85.0 (SE 5.9), 102.8 (SE 8.2); total fiber 92.8 (SE 5.2), 88.7 (SE 8.3); protein 106.2 (SE 8.3), 92.1 (SE 8.0); fat 102.6 (SE 8.3), 97.6 (SE 12.0); saturated fatty acids 102.1 (SE 14.0), 89.3 (SE 12.0); sodium 104.7 (SE 8.1), 105.7 (SE 10.8). Additional nutrients not available in the Tap & Track program were analyzed for Nutrihand compared to GRAND: cholesterol 99.8 (SE 9.2); vitamin A 75.3 (SE 15.7); vitamin C 119.1 (SE 17.0); calcium 126.2 (SE 30.0); and iron 97.3 (SE 5.5).

**Figure 3:**
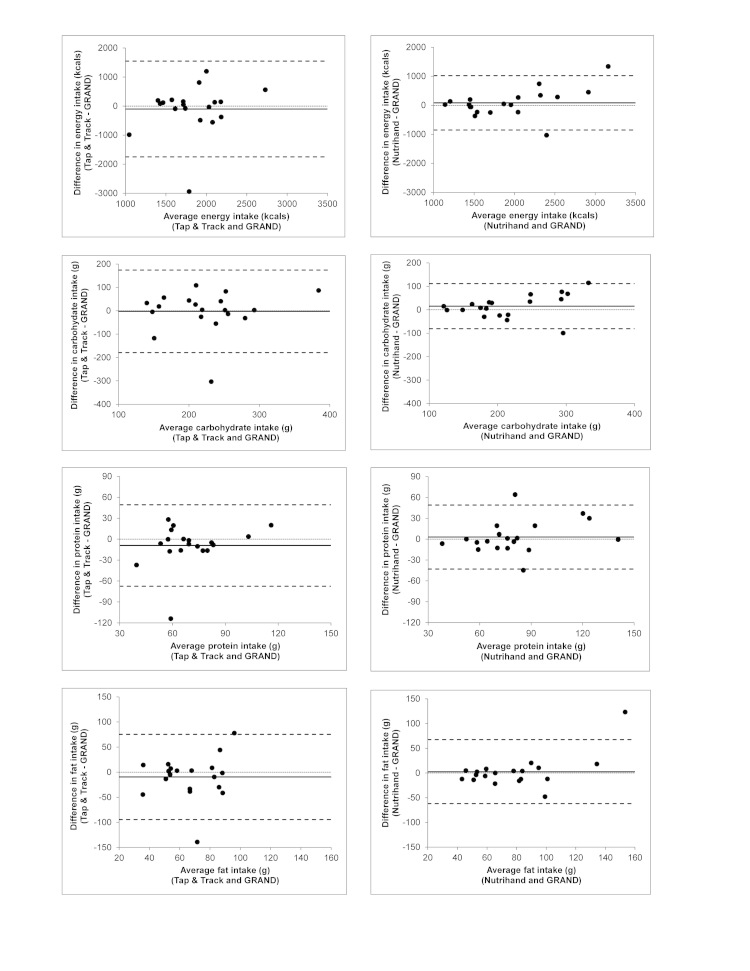
Bland-Altman plots comparing electronic diet entry by participants to dietitian entry of 3-day diet record (DR) into Grand Forks Research Analysis of Nutrient Data (GRAND). Plots for energy and macronutrients comparing Nutrihand and Tap & Track to GRAND. Solid horizontal line indicates mean of differences between Nutrihand or Tap & Track and GRAND. Upper and lower limits of agreement (dashed lines) define range within which most differences between methods are expected to occur. Dotted line at y=0 is given for reference.

The 95% limits of agreement of the electronic capture methods of diet recording compared to the dietitian-entered records are shown in Table 2. A statistically significant difference in mean reported intake was observed for sugars when comparing Nutrihand to GRAND. The Bland-Altman plots for nutrient estimates between the electronic diet capture and GRAND are illustrated in Figure 3 and Multimedia Appendices 1 and 2. The Bland-Altman plots display the variability of responses for each individual, for each nutrient evaluated for the two electronic diet methods under evaluation. On each plot, the mean difference from the GRAND estimates is illustrated as well as variance in individual responses.

**Figure 4:**
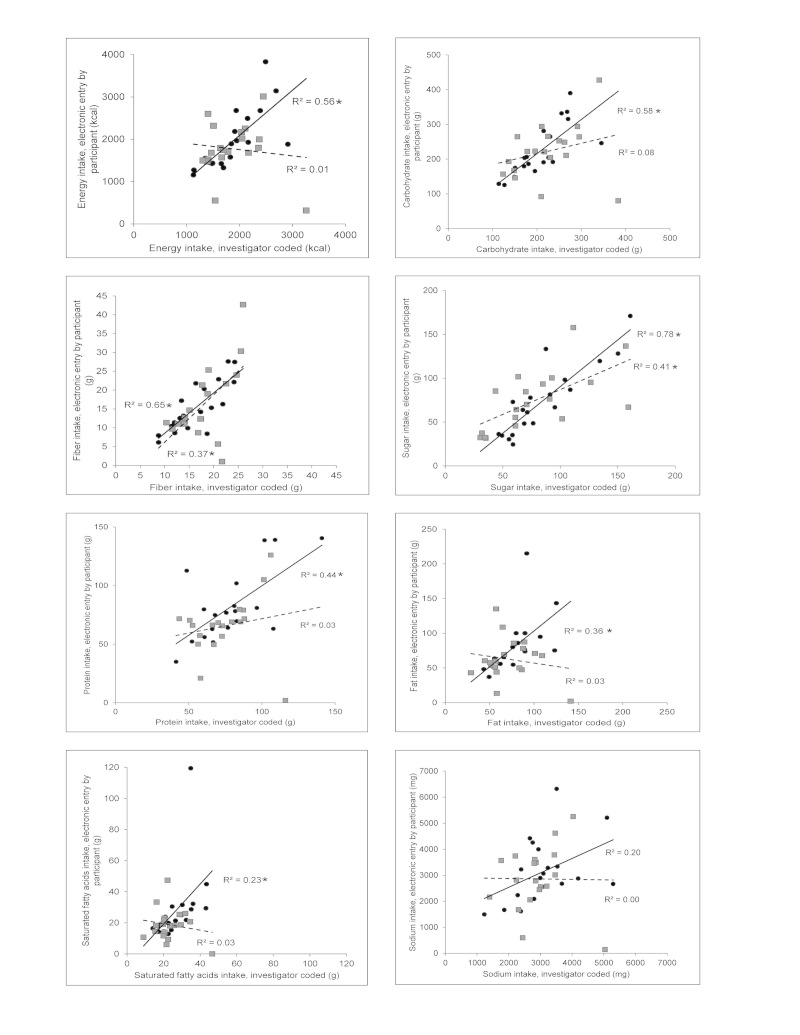
Plots comparing nutrient intakes estimated from 3-day diet records coded by investigator to same 3-day diet records entered electronically: Comparing Nutrihand (black circle) or Tap & Track (grey square) to Grand Forks Research Analysis of Nutrient Data (GRAND). Each point represents mean of food records for 3 days for each individual (n=19). Regressions comparing intake estimates from Nutrihand (solid line) and Tap & Track (dashed line) to estimates obtained from investigator-coded records were performed and R2 values are reported. *Statistical significance at P<.05.

**Figure 5:**
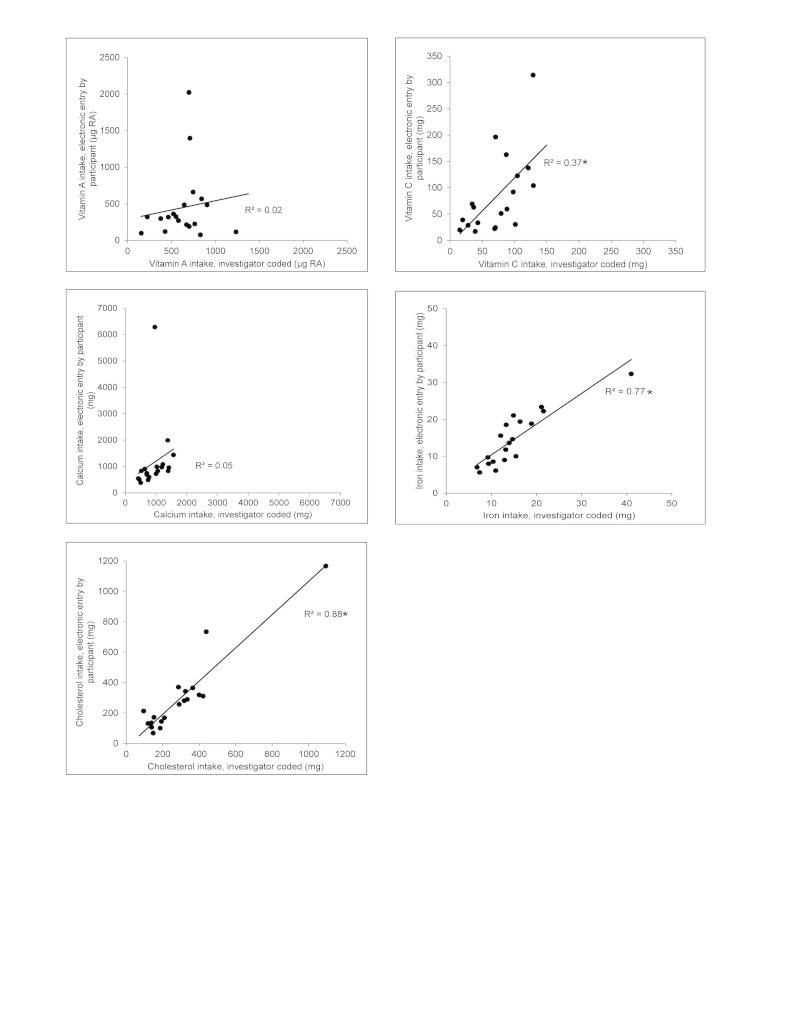
Plots comparing nutrient intakes estimated from 3-day diet records coded by investigator to the same 3-day diet records entered electronically: Comparing Nutrihand (black circle) to Grand Forks Research Analysis of Nutrient Data (GRAND). Each point represents mean of food records for 3 days for each individual (n=19). Regressions comparing intake estimates from Nutrihand (solid line) and Tap & Track (dashed line) to estimates obtained from investigator-coded records were performed and R2 values are reported. *Statistical significance at P<.05.

Correlation of the electronic diet assessment methods to the records coded and analyzed by the dietitian in GRAND are illustrated in Figures 4 and 5. Coefficients of determination, (*R* ^2^), for Nutrihand and Tap & Track compared to GRAND, respectively, were energy .56, .01; carbohydrate .58, .08; total fiber .65, .37; sugar .78, .41; protein .44, .03; fat .36, .03; saturated fatty acids .23, .03; and sodium .20, .00. Additional nutrients not available in the Tap & Track program were analyzed for Nutrihand compared to GRAND: cholesterol .88; vitamin A .02; vitamin C .37; calcium .05; and iron .77.

**Table 1:** Reported nutrient intake for GRAND and Nutrihand, and GRAND and Tap & Track.

Nutrient	GRAND	Nutrihand	GRAND	Tap & Track
	mean (SD)	mean (SD)	mean (SD)	mean (SD)
Energy (kcal)	1876.1 (501.1)	1961.4 (715.7)	1873.6 (499.4)	1772.9 (619.6)
Carbohydrate (g)	209. 1 (58.5)	224.6 (75.5)	224.8 (70.5)	222.3 (78.6)
Sugars, total (g)	85.1 (33.5)	74.7 (40.3^a^)	78.3 (39.3)	74.8 (34.8)
Protein (g)	79 (24.3)	82.1 (30.9)	74.6 (19.8)	65.5 (26.2)
Fat (g)	77.4 (23.9)	79.9 (41.3)	71.9 (26.4)	62.4 (30.1)
Saturated fatty acids (g)	27.1 (8.7)	28.3 (23.5)	25.4 (8.3)	18.7 (10.3)
Monounsaturated fatty acids (g)	26.7 (9.3)	24.3 (13.4)		
Polyunsaturated fatty acids (g)	15.3 (4.8)	11.3 (5.7)		
Cholesterol (mg)	298 (221.7)	298.5 (258.7)		
Total Fiber (g)	16.4 (5)	15.4 (6.6)	17.9 (4.7)	16.1 (9.7)
Calcium (mg)	930.1 (359.7)	1146.2 (1297.1)		
Iron (mg)	14.9 (7.5)	14.5 (7.2)		
Sodium (mg)	3107 (997)	3150 (1250)	2894 816	2859 (1239)
Vitamin A (mcg)	635.6 (248.9)	450.2 (482.9)		
Vitamin C (mg)	71.8 (37)	83.4 (76.5)		

^a^ *P*<.05 compared to GRAND by mixed model analysis of variance.

**Table 2:** Mean difference and limits of agreement^a^ between electronic diet recording (Nutrihand and Tap & Track) and dietitian-entered handwritten records (GRAND).

Comparison		Mean difference	95% Limits of Agreement	
			Lower limit	Upper limit
**Nutrihand to GRAND**				
	Energy (kcal)	85.3	−851.5	1022.1
	Carbohydrate (g)	15.4	−80.6	111.4
	Sugars (g)	−10.5^b^	−47.5	26.6
	Fiber (g)	-1.0	−8.7	6.7
	Protein (g)	3.1	−42.8	49.0
	Fat (g)	2.6	−62.4	67.5
	Saturated fatty acids (g)	1.3	−39.6	42.1
	Sodium (mg)	43.3	−2319.9	2406.4
	Cholesterol (mg)	0.6	−182.1	183.3
	Vitamin A (mcg)	−185.4	−1192.1	821.3
	Vitamin C (mg)	11.6	−109.1	132.3
	Calcium (mg)	216.0	−2265.5	2697.5
	Iron (mg)	−0.4	−7.6	6.8
**Tap & Track to GRAND**				
	Energy (kcal)	−100.6	−1748.7	1547.5
	Carbohydrate (g)	−2.5	−178.8	173.8
	Sugars (g)	−3.5	−65.8	58.8
	Fiber (g)	−1.8	−17.1	13.5
	Protein (g)	−9.1	−67.5	49.3
	Fat (g)	−9.6	−94.5	75.3
	Saturated fatty acids (g)	−4.7	−32.7	23.3
	Sodium (mg)	−35.1	−2959.1	2889.0

^a^The upper and lower limits of agreement define the range within which most differences between the methods are expected to occur.

^b^ *P*<.05 by mixed model analysis of variance.

## Discussion

### Principal Findings

Classically, the methods for assessing nutrient intake include the hand recording of intake on multi-day DR by participants, interviewer-assisted recalls, and diet histories or completion of paper questionnaires for food frequency assessment [5]. Use of computerized methods for dietary analysis by the dietitian/nutritionist came into common use in the early 1980s as personal computing became common in clinical dietetics practice [18,19]. Although these methods saved considerable time over hand computation, the time demands on the interviewer and analytic staff are still considerable. Recently, there has been an increase in the study of new technologies to reduce the cost of gathering dietary intake data [20,21]. Moreover, innovative technological methodologies are being investigated to improve the quality and completeness of dietary data gathered by electronic capture [8,10]. Computers and cell phones are commonly used by all age and sex categories and there is widespread availability and popularity of apps to track dietary intake [22].

A number of electronic methods of DR capture have been evaluated, although, to our knowledge, this is the first assessment of Web-based DR tools or apps available commercially for the public. Comparisons have been made between the accuracy of diet recording on paper or on computer and personal digital assistant (PDA) based software programs. Beasley et al compared diet records kept by a PDA software program, and hand-recorded records to a 24-hour dietary recall and concluded that the PDA-based program did not appear to be more valid than the hand-recorded records [23]. PDA-based software for dietary self-monitoring over 7 days was not found to improve energy intake estimates [24] or to provide comparable estimates [25] in reference to handwritten records. In a long-term study, Burke et al demonstrated that diet record keeping over 6 months was improved by PDA-based entry of records versus hand entry in a group of participants in a weight loss trial, but no assessment was made of program validity [26].

In comparison to dietitian-entered 3-day DR, the electronic methods evaluated herein resulted in no significant difference in mean estimates of macronutrients, but significantly lower estimates of most micronutrients. There was high concordance of mean values for reported nutrient intakes from the electronic capture compared to the dietitian entry of records, with the exception of sugars comparing Nutrihand to GRAND. However, when correlating individual values, the electronic methods resulted in point estimates with much larger variability, particularly with the use of Tap & Track. The Bland-Altman plots illustrate that, in general, the responses obtained from Tap & Track displayed more variability than those from Nutrihand indicating reduced agreement. When a dietitian reviews diet records with participants and clarifies issues of food items, portion size, and added condiments prior to entry into a nutrient analysis program, the intake estimates are improved. The greater variability of nutrient estimates we observed with the electronic capture methods may be due to factors associated with diet record-keeping accuracy, including food options in the database, and the ability of participants to pick appropriate food items, to estimate portion sizes, and to accurately account for all foods consumed [22].

Reported energy intake in this study was less than that found in the What We Eat in America, National Health and Nutritional Examination Survey (WWEIA, NHANES 2009-2010) for men of this age (2482, SD 55.3 kcal), but similar to that found in women (1759, SD 38.4 kcal) [27]. This is in contrast to recent research comparing interviewer-coded DR to the Web-based, participant-coded Automated Self-Administered 24-hour recall (ASA24), which found much lower energy estimates [6]. This may be a reflection of the design differences in the investigation. Typically, diet recording is done concurrently or close to the time of food consumption, which may reduce the error seen in a diet recall performed the day after intake.

Although lower mean intake values for many micronutrients were reported with Nutrihand compared to GRAND, dietary fiber, calcium, potassium, and sodium, and other evaluated nutrients were concordant. A major methodological difference is that the hand-recorded DR method is open-ended; any food may be recorded, with detail. When using the electronic methods of DR entry, the foods are limited to those in the database. Recording errors such as implausible amounts of food consumed or foods lacking detail enough to code can be resolved during the dietitian review. During the coding process, the dietitian is better-suited to make informed decisions about coding combination foods or foods without brand names than the client [23]. It appears that dietitian-entered DR are most appropriate when the data collected is a primary research outcome and when micronutrient intake are required. However, if the goal of the DR is to collect macronutrient data as a secondary outcome variable or to allow clients/study participants to monitor their own nutrient intake, then electronic capture of DR with Nutrihand or Tap & Track may be appropriate.

The paper-based, handheld iPod and Web-based methods may be recorded at different times relative to eating. While the DR and Tap & Track can be entered immediately after eating or drinking, a client must wait until they have computer and Internet access to record with Nutrihand. As DR are the fastest way to record what is eaten, it is probable that the DR was used as a guide when entering Nutrihand, which may explain some of the smaller variability compared to Tap & Track. Despite these cognitive differences, the three methods were remarkably similar.

### Strengths and Limitations

One of the strengths of our project was the fact that we randomly assigned participants to the order of the electronic DR capture during the two washout periods of the clinical trial. Another is that all participants were provided detailed instruction by the dietitian in how to maintain a complete and accurate diet record. Due to the similarity of energy intake of the DR analyzed in GRAND compared to the nationally representative WWEIA, NHANES data, it appears that participants were able to provide estimates of usual intake. These results are therefore appropriate for use as the standard with which to compare the electronic dietary recording methods. An additional strength of the study is our use of a highly rated app (Tap & Track) for the handheld record, indicating that the app is popular and easy to use.

We were limited by the small sample size of the study; however, the large number of DR allowed us to statistically test differences between methods. In retrospect, it may have been appropriate for us to include a non-correlated dietary intake method in our study design as between-method agreement is enhanced when the source of measurement error is correlated. There are limitations in the use of the Bland-Altman analysis for the comparison of these methods of diet assessment. These analyses are based on the assumption that measures are performed on the same samples. However, it is possible that those individual foods entered by participants into the electronic diet-recording device and those entered by the dietitian into GRAND may have been different. Nevertheless, this assessment allows us to estimate the amount and direction of variability in responses. All self-reported diet assessment is subject to error and bias. A participant may choose to over-report foods perceived as healthy or not report foods perceived as unhealthy. If it is not convenient, a participant may not record food items immediately and may forget eating them. Portion size is difficult to determine and may be under- or over-reported. Even the act of recording foods consumed can change a person’s eating habits, either purposefully or not. A potential limitation of this study is that the errors in the different methods may be correlated, which would increase observed correlations.

There is a need for further development of valid digital methods of dietary assessment. These must be simple for participants to use and not overly burdensome for dietitians and researchers to interpret. Disposable cameras have been used to capture meal images before and after consumption, but are inconvenient for use and did not provide enough information for interpretation. Much research has focused on digital camera images. Digital cameras are more convenient for participants, but still suffer from lack of information for use without having participants provide additional information. Both also need to be coded by a dietitian. Currently, Web-based and mobile phone diet entry may be considered to be, on balance, useful tools that minimize both participant and researcher time [20-22].

### Conclusions

This study demonstrated that two readily available self-administered electronic DR capture methods (Nutrihand and Tap & Track for the iPod) showed high concordance of mean nutrient values with a traditional dietitian-coded and analyzed DR. These data indicate that electronic diet recording may be suitable for group intake estimates and their use may reduce time spent on dietary assessment. Compared to the dietitian-coded and analyzed DR, Nutrihand in particular performed well, especially for energy and macronutrients, both critical in self-monitoring of dietary intake. Our results suggest that electronic DR capture may be appropriate for diet monitoring and useful in reducing the workload of DR coding and entry. The validity of nutrient intake estimates by electronic capture for an individual needs further assessment. Additional research is required to evaluate other electronic DR capture methods as is work to improve the precision with which clients are able to enter their dietary intake.

## Multimedia Appendix 1

Bland-Altman plots comparing electronic diet entry by participants to dietitian entry of 3-day diet record (DR) into Grand Forks Research Analysis of Nutrient Data (GRAND). Plots for sugars, fiber, SFA, and sodium comparing Nutrihand and Tap & Track to GRAND. Solid horizontal line indicates mean of differences between Nutrihand or Tap & Track and Grand. Upper and lower limits of agreement (dashed lines) define the range within which most differences between methods are expected to occur. Dotted line at y=0 is given for reference.

## Multimedia Appendix 2

Bland-Altman plots comparing electronic diet entry by participants to dietitian entry of 3-day diet record (DR) into Grand Forks Research Analysis of Nutrient Data (GRAND). Plots for cholesterol, vitamin A, vitamin C, calcium, and iron comparing Nutrihand to Grand. Solid horizontal line indicates mean of differences between Nutrihand or Tap & Track and Grand. Upper and lower limits of agreement (dashed lines) define range within which most differences between methods are expected to occur. Dotted line at y=0 is given for reference.

## Abbreviations

ARS: Agricultural Research Service
DR: diet records
FIAS: Food Intake and Analysis System
GFHNRC: Grand Forks Human Nutrition Research Center
GRAND: Grand Forks Research Analysis of Nutrient Data
SR22: National Nutrient Database for Standard Reference, Release 22
USDA: United States Department of Agriculture
